# Potential biomarkers to predict return to fertility after discontinuation of female contraceptives—looking to the future

**DOI:** 10.3389/frph.2023.1210083

**Published:** 2023-08-22

**Authors:** Amanda Cordova-Gomez, Andrew P. Wong, Lee B. Sims, Gustavo F. Doncel, Laneta J. Dorflinger

**Affiliations:** ^1^ Office of Population and Reproductive Health, USAID/Public Health Institute, Washington, DC, United States; ^2^ CONRAD, Department of Obstetrics and Gynecology, Eastern Virginia Medical School, Norfolk, VA, United States; ^3^ Department of Product Development and Introduction, FHI 360, Durham, NC, United States

**Keywords:** contraception, return to fertility, biomarker, hormonal, pregnancy, folliculogenesis, ovulation, progesterone

## Abstract

Nowadays there are multiple types of contraceptive methods, from reversible to permanent, for those choosing to delay pregnancy. Misconceptions about contraception and infertility are a key factor for discontinuation or the uptake of family planning methods. Regaining fertility (the ability to conceive) after contraceptive discontinuation is therefore pivotal. Technical studies to date have evaluated return to fertility by assessing pregnancy as an outcome, with variable results, or return to ovulation as a surrogate measure by assessing hormone levels (such as progesterone, LH, FSH) with or without transvaginal ultrasound. In general, relying on time to pregnancy as an indicator of return to fertility following contraceptive method discontinuation can be problematic due to variable factors independent of contraceptive effects on fertility, hormone clearance, and fertility recovery. Since the ability to conceive after contraceptive method discontinuation is a critical factor influencing product uptake, it is important to have robust biomarkers that easily and accurately predict the timing of fertility return following contraception and isolate that recovery from extrinsic and circumstantial factors. The main aim of this review is to summarize the current approaches, existing knowledge, and gaps in methods of evaluating return-to-fertility as well as to provide insights into the potential of new biomarkers to more accurately predict fertility restoration after contraceptive discontinuation. Biomarker candidates proposed in this document include those associated with folliculogenesis, cumulus cell expansion, follicular rupture and ovulation, and endometrial transport and receptivity which have been selected and scored on predefined criteria meant to evaluate their probable viability for advancement. The review also describes limitations, regulatory requirements, and a potential path to clinically testing these selected biomarkers. It is important to understand fertility restoration after contraceptive method discontinuation to provide users and health providers with accurate evidence-based information. Predictive biomarkers, if easy and low-cost, have the potential to enable robust evaluation of RTF, and provide potential users the information they desire when selecting a contraceptive method. This could lead to expanded uptake and continuation of modern contraception and inform the development of new contraceptive methods to widen user's family planning choices.

## 1. Introduction

Over a dozen types of modern contraceptive methods, from reversible to permanent, are available for individuals with the potential to become pregnant, but who choose to delay pregnancy (1). Multiple new technologies are being developed to expand the contraceptive method mix, filling gaps in current options and appealing to a diverse set of end-user preferences (2–4). Although expanded access to contraceptives has improved, the proportion of women of reproductive age that have their family planning needs met has only increased from 73.6% in 2000 to 76.8% in 2020 globally (5). Barriers to access and use of contraceptive methods remain, with an estimated 225 million women in low- and middle-income countries (LMIC) experiencing unmet need for modern contraception (6).

For users wanting to become pregnant, recovering the ability to conceive after discontinuing their use of a modern contraceptive i.e., having fertility return, is pivotal (7). Furthermore, the stigma behind being infertile is a universal trend, especially in LMIC contexts where fertility impairment still constitutes a social, psychological, and economical burden for gender women (8).

Beliefs around infertility and family planning use are interconnected (9), and the perceived ease or difficulty of being able to conceive after discontinuing contraception plays a notable role in the decision to initiate and continue contraception (10). For example, in one study among married females in LMIC wishing to avoid pregnancy but not using contraception, 26% cite side effects and other health concerns, including infertility, as reasons for non-use (6, 11). Among youth specifically, one of the key fears about hormonal contraception is indeed a delayed return to fertility (RTF) or fear of permanent infertility (12, 13).

A number of studies have assessed RTF following discontinuation of hormonal contraceptives, but most date from about 10 to 20 years ago. Results are variable based on methodology, and across method type, with median rate of pregnancy achievement ranging from less than 6 months to 12 months (14–18). While the primary assessment of time to RTF in some earlier studies was calculated retrospectively based on recruiting women at the time of pregnancy achievement/birth (16, 17, 19), others followed women prospectively until conception (14, 15), while others prospectively evaluated return to ovulation as a surrogate for RTF (20–22), measuring levels of progesterone, luteinizing hormone (LH), and follicle-stimulating hormone (FSH) (23), or a combination of these with or without transvaginal ultrasound (24).

In general, relying solely on time to conception as an indicator of RTF following contraceptive method discontinuation can be problematic due to variable influences independent of contraceptive effects on fertility, hormone clearance, and fertility recovery. The achievement of pregnancy will depend also on the user's behaviors and experiences (25), sexual frequency and timing (26), and the user's and the partner's underlying fertility and intention to become pregnant. Furthermore, genetic, behavioral, and environmental factors might potentially affect fertility and pregnancy post-discontinuation of contraception, and may differ among regions (27). Additionally, RTF following discontinuation can be influenced by factors such as lifestyle (28), body mass index (BMI) (29), age (30), menstrual cycle characteristics (31), and reproductive tract health (32), among others (33).

Since the ability to conceive after contraceptive method discontinuation is a critical factor influencing user uptake, it is important to have robust biomarkers that easily and accurately predict the timing of fertility return following contraception and isolate that recovery from extrinsic and circumstantial factors. The exploration of new accurate and reproducible biomarkers [for a definition please see (34)] is pivotal to evaluate not only new contraceptive products but also potentially to confirm RTF among users of methods with inherently highly variable durations (e.g., injectables). Those who seek to monitor the preservation of their fertility after cancer treatment may also benefit from potentially more robust indicators.

The main aims of this review are to summarize the current approaches, existing knowledge, and gaps in methods of evaluating RTF as well as to provide insights into the potential of new biomarkers to efficiently and effectively understand and predict fertility restoration after contraceptive discontinuation. It is important to understand fertility restoration after contraceptive method discontinuation to provide users and health providers with accurate evidence-based information. Predictive biomarkers, if easy and low-cost, have the potential to enable robust evaluation of RTF, and provide potential users the evidenced-based information they desire when selecting a contraceptive method. This could lead to expanded uptake and continuation of modern contraception and inform the development of new contraceptive methods to widen user's family planning choices (35).

## 2. The need for further return to fertility biomarkers

The regulatory requirements for oral contraceptive approvals by the U.S. Food and Drug Administration (FDA) were initially the same as any other drug (36). Then, in the 1960s, as a result of the tragedy surrounding thalidomide and the observation of mammary tumors in dogs following treatment with some progestins, the FDA adopted stringent safety criteria for preclinical and clinical studies for steroid-based contraception (36) although clinical studies on RTF were not strictly required. In the late 80's, the impact of treatment on fertility in animals, and the possible effects of the drug in progeny, were included as part of required reproductive toxicology studies (37, 38) based on the recommendation from a WHO symposium on Safety Requirements for Contraceptive Steroids. These evaluations focus on reproductive and developmental toxicity as well as return to fertility in animal models (36, 38), However, these non-clinical studies did not address or inform the question of timing of RTF in humans. Therefore, studies evaluating the effect of contraceptives (older as well as newer products) on RTF were and are still limited.

Many studies on RTF following use of combined oral contraceptives (COCs) containing both an estrogen and progestin are more than 20 years old and provide data on products or formulations that are different or no longer used today (17, 19). A study from 1978 suggested a temporary impairment of fertility after discontinuation of 50 ug estrogen (ethinyl estradiol, EE) oral contraception, independent of the duration of use, but not a permanent fertility impairment (17), and a cross-sectional study in 1982 measuring conception at 1-year after cessation of contraception found a return to a fertility rate of 72% (19). In contrast, research published in 2005 (16) reported that in a community-based population of fertile women aged 20 to 40 years, the time to pregnancy was affected by type of previous contraception used. Maternal age was reported to have a significant impact on time to conception in two of these studies (16, 19). Other than age, the reason for any potential transiently impaired fertility found in the different studies is not well understood (39). The longer delays found in early studies compared to other more recent studies might be due to the higher doses of oral contraception used in the 1980s in addition to different methodologies (retrospective vs. prospective studies) and data collection after delivery.

Studies evaluating the impact of length of COC use on RTF measured different indicators of fertility and found varying results. A retrospective study in 2005 with 680 women, found that after COC discontinuation, there was no difference in the length of the menstrual cycle between users and non-users independently of the COC dose (40). A different study (41) found a progressive decline in fertility with prolonged COC use, measured by time to pregnancy after cessation of use, while yet another (42) found improved fertility measured as time to conception after extended use of COCs (41). Methodological differences exist among the different studies, such as the exclusion of short-term users, the number of participants, age of the cohort of individuals using long-term contraception, as well as the absence of appropriate controls, among others. All these various methodological differences could contribute to these studies having different results. Moreover, the length of the menstrual cycle can be affected by different factors (43), and can be very variable among individuals as determined previously by several other studies (44, 45).

In 2006, a study with 750 women (46) reported a one-year conception rate of 86.6% (613 women) after discontinuation of COC [30 ug EE + 2 mg dienogest (DNG)]. The main limitations of this research, as seen in some other studies using conception rate over time as the ultimate indicator of fertility, is a lack of information about the partner, frequency of intercourse, lifestyle, time, and the potential use of other contraceptives after EE/DNG use. In this retrospective study, only women who achieved pregnancy were included. Women who failed to become pregnant or who gave up attempting pregnancy were excluded, likely causing a skewed return to fertility estimation (47). While a retrospective study assessing time to pregnancy can be conducted at lower cost than prospective studies, the design can also bring biases and assumptions when there is a lack of detailed information on exposure and behavior (47, 48). Some studies have also demonstrated that COC use may affect the first cycles after discontinuation (16, 49, 50) but without having a long-lasting detrimental effect as 1-year pregnancy rates are not altered (7). However, despite the variable results of these earlier studies related to potential delays in RTF after COC use, the more recent large prospective multinational, multicenter study of different formulations of COCs (EURAS-OC) (51), and a latest systematic review of evidence in 2018 (7), found no negative effect after 1 year of COC use on time to conception, regardless of the duration and type of COC.

RTF after depot-medroxyprogesterone acetate (DMPA) injectable discontinuation was also assessed in early studies. One study (52) in 1973, followed 135 women stopping use of DMPA (150 mg intramuscular injection, every 12 weeks), to become pregnant and concluded that, RTF (measured as pregnancy achievement) 14 months after contraceptive discontinuation was just slightly lower than those women discontinuing COC or IUDs. A 1980 study (14) included 796 Thai women who stopped DMPA use and 125 removing their IUD due to pregnancy intentions. The median delay to conception was 5.5 months for DMPA users (plus the duration of DMPA effects of the last injection: around 15 weeks) and 4.5 months for IUD users. This study was compared to a smaller study (15) where the pregnancy rate among users discontinuing norethisterone enanthate (NE) was not significantly different from non users, or from those not using nonsteroidal contraception after NE was cleared from the body.

Several studies have evaluated RTF following discontinuation of contraceptive implants, with the one-year pregnancy rate following implant removal (ranging from 76 to 100%) having been the primary indicator of a RTF (53). Norplant and Norplant-2, both of which are no longer commercially available, provided sustained release of low dose levonorgestrel (LNG). In 1990, product developers began work on a reformulated two-rod LNG releasing system, now commercially available as Jadelle. The 1995 study by the ICMR Task Force on Hormonal Contraception followed 627 women who discontinued Norplant-II for up to 2 years (54) and reported that 20% of women became pregnant within one month of implant removal. The cumulative pregnancy rate was 63.4% at 6 months, 80.8% at one year, and 88.3% at 2 years. Researchers also observed that RTF was significantly more delayed in those women older than 30 years old, whose cumulative pregnancy rate was 66.3% at one year with a median time to conception of 6 months (vs. 3.8 months for women 30 years old or younger) (54). The observed differences of pregnancy rates among older vs. younger women in this study could be due to a variety of confounding behavioral and physiologic factors, as discussed above. This further substantiates the problematic nature of relying solely on pregnancy rates following contraceptive discontinuation as a marker of fertility restoration.

In a five-year comparison of clinical outcomes between the use of Norplant implants and a copper IUD, Singh et al. observed 11 out of 14 (78.5%) participants who discontinued Norplant conceived within one year following discontinuation (55). At two years post-discontinuation, 75 out of 78 ex-Norplant users conceived (55). Few studies have evaluated external factors on pregnancy achievement following discontinuation of LNG releasing implants. However, a multicenter, prospective study investigating rates and outcomes of planned pregnancies following Norplant and Norplant-II discontinuation observed that age at implant removal and family planning intentions upon study enrollment were both significantly correlated with pregnancy achievement rates (56). The study found that participants whose age was less than 30 at the time of implant removal had significantly higher pregnancy rates relative to participants 30 years or older (56). Additionally, those participants who entered the study with the intention of using family planning as a method of spacing pregnancies also had a significantly higher rate of pregnancy achievement after discontinuation relative to those whose initial intentions were to limit pregnancies (56). This study highlights the underlying physiologic and behavioral factors that can contribute to the rate of pregnancy achievement following contraceptive discontinuation, underscoring the need for more robust and independent methods of evaluating fertility restoration.

In more recent years, meta-analyses and systematic reviews assessing fertility following contraceptive discontinuation have been performed. One such review used 12-month conception rates as the primary metric of evaluating RTF and conducted a meta-analysis pooling 22 studies, including a total of 14,884 users who discontinued contraception in the final analysis. Of these, the pooled 12-month pregnancy rate was 83.1% (95% CI = 78.2%–88%). Investigators did not find significant differences in 12-month pregnancy rates between hormonal methods and IUD (copper or hormonal IUD; mean of 84.75%) (7). More recently it was reported that 86.1% of women who discontinued a newer hormonal IUD to attempt to conceive did so within 12 months (57). In a multivariable analysis, there was no difference in rates among nulligravid and gravid women; only obesity impacted ability to conceive. In studies of a recently approved combination vaginal ring, Annovera, RTF was assessed in 290 of the participants in the Phase III trials who desired pregnancy or switched to another non-hormonal contraceptive method after the trial. All participants noted a RTF within the 6 months, determined by pregnancy achievement or return to menses (58).

The above overview focuses on pregnancy as the outcome defining RTF and highlights the challenges as well as the mitigating factors that influence pregnancy achievement. Understanding the mechanisms by which contraceptive methods work is essential to subsequently evaluating RTF following use. Biomarkers that predict the ability to conceive have been used to study hormonal approaches that act, at least in part, through suppression of ovulation.

## 3. Importance for contraceptive research and development

As discussed above, RTF following discontinuation of contraceptive methods has historically been assessed by evaluating pregnancy rates over a designated period such as one year among women who discontinue contraceptive method use specifically to seek pregnancy (7, 27). For new contraceptive products, the current European Medicines Agency (EMEA) Guideline on Clinical Investigation Of Steroid Contraceptives In Women states that developers should follow all individuals in pivotal registration trials for up to a year to assess time to return of fertility if they discontinue specifically for desired pregnancy (59). The U.S. Food and Drug Administration (FDA) does not currently have a similar guideline, however, in Class Labeling for Combined Oral Contraceptives, product developers are asked to report data on RTF following discontinuation of product use, if available (60). A key challenge to rigorously assessing RTF following use of investigational products during the development phase is one of numbers: typically, only small numbers of Phase III study participants discontinue for desiring pregnancy and are willing to continue follow-up. In addition, as discussed above, many factors unrelated to method use can confound this assessment of RTF when using pregnancy as the outcome. For methods that suppress the hypothalamic-pituitary-ovarian axis and inhibit ovulation as their primary mechanism of action, evaluating return to normal ovulatory function has been used as a marker of potential fertility. The EMEA Guideline notes that “the time to return to normal ovarian function after discontinuation” should be studied in a sufficient number of patients (59). Numbers considered “sufficient” are not provided nor is there a recommended approach for assessing “normal ovarian function”.

The following sections will detail what is known about biomarkers and describe the potential biomarkers, as well as the research gaps that must be filled such that new, robust, and easier ways of evaluating RTF after discontinuation can be applied during product development.

## 4. Biomarkers currently used to indicate fertility

### 4.1. Assessing ovulation as a biomarker

Regulation of follicular development, ovulation and luteal function is complex and multifactorial (61). Careful characterization of ovulation is done by frequently-timed transvaginal ultrasonography to measure follicular development and rupture and corpus luteum development, coupled with measuring blood levels of progesterone as well as estradiol, LH, and FSH. A six-point grading system was developed by Hoogland and Skouby in the early 1990s to characterize ovarian activity from “no activity” to “ovulation” based on follicular rupture, plus estradiol and progesterone, and has been widely used (24). This approach is sometimes modified by investigators based on research objectives. For example, research to quantify ovarian activity and ovulation among combined or progestin-only pill users have considered stratified luteal phase progesterone levels in the algorithm i.e., whether progesterone levels following rupture of a lead follicle were considered adequate to confer a theoretical risk of pregnancy (62–64). While evaluating multiple factors involved in the ovulatory process has been the gold standard for concluding an ovulation has occurred and for assessing whether it is functional, the demand on both participants and researchers is great. More commonly, presumptive ovulation has been assessed without using ultrasound, measuring only serum or urinary hormones i.e., progesterone (or progesterone plus estradiol) (65–68), urinary pregnanediol—which has been shown to reflect serum progesterone (69, 70), and sometimes including LH and FSH levels (64, 71, 72).

With regard to using progesterone as a biomarker of ovulation, detailed assessments of daily serum levels across the entire menstrual cycle have been published (69, 73) and the range of luteal phase progesterone values considered to be “normal” after ovulation (determined by observation of an LH peak) classified based on median, 5th and 95th percentile values. Many studies in the literature use more limited sampling, however, and the varied timing and frequency of sample collection as well as the differing cut-off levels of progesterone used to conclude an “ovulation” has occurred make precise comparisons difficult. Some definitions will lead to more liberal determinations when classifying a presumptive ovulation i.e., those that use a single low progesterone value. A recent study documented the variability in concluding a presumed ovulation when using several different progesterone level cut-offs (68). Finally, progesterone values on the low end of normal that have been considered “ovulations” in many studies e.g., near the 5th percentile, may not be functional ovulations indicative of “fertile cycles”. A recent review summarized the challenges with using progesterone alone when classifying normal or deficient luteal phases (74). However, research on pregnancy success following timed intercourse or intrauterine insemination reported that midluteal levels of progesterone were significantly lower in cycles where pregnancy was not achieved compared with cycles where pregnancy was achieved (75, 76).

### 4.2. Influencing factors and considerations

With all hormonal methods, wide interindividual variability in steroid levels and responses are observed, and understanding is limited with regard to the impact of this variability on method-specific contraceptive effectiveness and side effects (65, 77–80). Pharmacogenomic differences, BMI differences, and ethnicity among other factors have been shown to influence pharmacokinetics and pharmacodynamics (81, 82). Evidence of the influence of stress on menstrual cycles and ovulation is also accumulating (83, 84). In addition, one study reported that the prevalence of luteal phase deficiency (low progesterone and shorten luteal phase) is more common in women with obesity during natural ovulatory cycles (85), adding further complexity to evaluating method-specific RTF based on ovulation (particularly progesterone alone) as a biomarker or surrogate marker.

Furthermore, fairly recent large population studies showed that about 1/3 of cycles with stated normal length (21–35 days) have subclinical ovulatory disturbances with low progesterone (68, 86). The authors speculate that anovulation periodically occurs in all or most women and note the need for more population-based studies to assess within-woman variability over time (68). With this as a backdrop, it may be challenging when using ovulation as a biomarker to determine precisely whether an observed delay in RTF following discontinuation of a method is truly method-related, or inherent in some variability of individual menstrual cycles.

Some highly effective hormonal methods, e.g., certain progestin-only pills (POPs), levonorgestrel implants, and the hormonal IUD, only partially suppress ovulation. Additional effects include altering the regulation of follicular development leading to anovulation, abnormal ovulation, inadequate luteal phase or a luteinized unruptured follicle (64, 66, 67, 71, 87, 88), reduced endometrial thickness (89), and progestin-induced thickening of cervical mucus causing it to become impenetrable to sperm (66, 88, 90–92). Cervical mucus quality is typically assessed using a modified Insler score as well as a sperm penetration assay (93). A review on the role of cervical mucus and contraception discusses the methodological limitations with these assays (91). However, a recent well-designed study of a levonorgestrel POP showed that cervical mucus quality did not favor fertility in users of this POP, even when a cycle was classified as ovulatory (88). This study is consistent with earlier studies on levonorgestrel implants (66) and helps to clarify the understanding of the important role of cervical mucus in the mechanism of progestin-only methods as well as its potential to influence RTF following use of certain progestin-only contraceptive approaches.

For methods where discontinuation of use leads to a rapid elimination of the circulating progestin (e.g., POPs, implants, hormonal IUD), the ability to conceive has been shown to resume without a lingering effect (61, 94). In contrast, injectable contraceptives that form drug depots e.g., medroxyprogesterone acetate or norethindrone enanthate, have a more variable and delayed return to ovulation and to fertility at the end of the period of high effectiveness (22, 65, 78) due to the continued impact of low, circulating levels of progestins and the mechanisms described above (66, 67, 71, 87, 88). This reinforces the concern that using only progesterone levels as a biomarker for putative ovulation may overestimate fertility potential if residual contraceptive progestin serum levels are detectable e.g., with injectable, non-removable approaches (22).

### 4.3. Biomarkers used in the infertility field

The contraception field can benefit by considering knowledge gained through decades of research related to infertility and perhaps leverage data on predictors of successful pregnancy following *in vitro* fertilization. A number of factors have been considered as biomarkers of ovarian reserves and as predictors of fertility or potential success in infertility treatment. These include antral follicle count (AFC), anti-Mullerian hormone (AMH), FSH, and InhibinB (95, 96). Whether any of these could potentially be used alone or combined with assessments of ovulation to more accurately predict RTF remains to be determined.

One study evaluated whether the use of long-term COC is associated with AFC suppression in patients seeking fertility preservation (FP) before cancer treatment (97). They reported that long-term use of COC is strongly associated with reversible AFC suppression, which returns to normal by 6–7 months after COC stoppage. This study agreed with other studies that found not only low AFC but also low AMH and ovarian volume during COC use (98, 99). However, greater precision in the timing of return to normal would be necessary to make these indicators useful. In addition, AFC and ovarian volume are determined through use of ultrasound that is more difficult than measuring a serum hormone such as AMH.

AMH is expressed by granulosa cells of growing follicles, is regulated by LH, FSH, estradiol and bone morphogenic proteins, and in turn regulates folliculogenesis through a variety of target genes (100). AMH is a marker of ovarian reserve that peaks around age 16, remains stable through the mid-20s and then declines with age (an inverse correlation with age). AMH levels have been shown to vary across the menstrual cycle; however, while statistically significant (with highest levels in the mid-follicular phase), changes across the cycle were small and not considered clinically important as related to timing of sampling for diagnostic purposes (100, 101). Investigators did note, however, that AMH levels were slightly higher in anovulatory cycles than in ovulatory cycles. Many studies have documented the inhibitory effect of estrogens on AMH during follicular growth, including one evaluation of the impact of continuous use of three types of estrogen-containing contraceptives (OCs, a vaginal ring and a patch) that noted significant declines (approximately 50%) in AMH after nine weeks of use in all groups (102). More recently, a retrospective cohort study assessed the impact of various contraceptives including COCs, a combined vaginal ring, POPs, the levonorgestrel IUD and non-hormonal methods on AMH and antral follicle count and reported decreases in AMH by COCs and POPs, but not the other methods (103). Another showed AMH levels to be markedly reduced among current users of COCs, a combination vaginal ring and DMPA, but not among current users of the levonorgestrel IUD (104). There was no significant association between AMH and time since last use for COC users, but AMH levels were lower among individuals who had used DMPA within the last year when compared to never users. Given DMPA's reduction in AMH in the study, the latter difference might be explained by the extended time for MPA levels to return to undetectable following a user's last injection (22, 78).

Several important factors need to be considered in assessing whether AMH might be used as an indicator of RTF, or as a component of other algorithms of RTF. These include the impact of various other influencers of AMH levels including age, race or ethnicity, BMI, and environmental or other factors that might confound result interpretation (100, 105). In addition, better information on the time course to return to a previous baseline, for example after COC discontinuation, would be necessary to assess operational questions such as whether an individual would need a baseline sample before initiating contraception in order to accurately assess recovery (RTF). Finally, in a prospective cohort study of 981 women without a history of infertility who were trying to conceive, the probability of conception was the same for women with low and normal serum AMH levels and the authors concluded that their findings do not support use of AMH to assess natural fertility or reproductive potential (106).

Another hormone that has been used to predict fertility potential in *in vitro* fertilization (IVF) programs is the pituitary hormone FSH. During a normal menstrual cycle, FSH levels rise during menses and stimulate granulosa cell production of estradiol and Inhibin B. Driven by the negative feedback of increasing estradiol and Inhibin B, FSH levels then decline by the mid-follicular phase (61). An FSH level on cycle day 3 has been considered a marker of ovarian reserves and has been a widely used test in the clinical evaluation of infertility. However, a review in 1999 concluded that “the evidence does not support the use of these tests for widespread screening of women for fertility potential” and “will result in an unacceptably large percentage of false-positive test results” if used in the general population (107). A 2006 systematic review similarly concluded that measurement of basal FSH in the early follicular phase has a limited capacity to predict IVF outcome, and therefore should not be routinely used for this purpose (95). More recently, in the prospective study of Steiner and colleagues noted above (106), women with high serum or urinary FSH (a potential indication of reduced ovarian reserves) did not have a significantly different probability of conceiving after 6 or 12 attempted cycles compared with women who had normal FSH values. They concluded that their data do not support the use of FSH to assess natural fertility. It follows, therefore, that an early follicular phase FSH level would not be a sensitive potential biomarker of RTF after discontinuation of contraceptives that inhibit FSH as part of their mechanism of action (contraceptives with an estrogen component).

Inhibin B belongs to the superfamily of transforming growth factor-B. It is secreted by granulosa cells and provides negative feedback at the level of the pituitary to inhibit FSH secretion during the normal menstrual cycle (108). Inhibin B is also considered one of the indicators of ovarian reserves that is used in the context of infertility assessment and treatment. As women age, Inhibin B levels gradually decline leading to progressive increases in basal FSH. While a number of studies have evaluated the use of basal Inhibin B, alone or in combination with other ovarian reserve indicators, as a predictor of success in infertility treatment, one systematic review and meta-analysis concluded that the predictive value is modest to poor (95). Although a recent study concluded that Inhibin B may have an advantage over FSH in predicting ovarian response to infertility treatments (109), another in a cohort of women *without* infertility noted above (106) concluded that Inhibin B levels were not associated with the probability of conceiving in a given cycle. Based on the composite of data, as with AMH and FSH, the use of Inhibin B as a potential biomarker of RTF following contraceptive use would not be recommended.

## 5. Promising new molecular biomarkers to predict RTF

While hormonal contraceptive approaches uniformly impact ovarian function and ovulation, and markers of such, the indicators for RTF following use of new contraceptives with novel mechanisms of action will depend on their mechanism of action. As such, there is a need to identify additional non-hormonal molecular biomarkers predicting RTF after method discontinuation. As noted above, extant measures of returning fertility, commonly revolving around pregnancy or ovulation resumption, face problems and take time to evaluate. Additionally, the exploration of contraceptive methods utilizing different mechanisms of action supports the identification and application of specific biomarkers associated with targets of these mechanisms, such as folliculogenesis, follicle rupture and ovulation, and endometrial transport and receptivity, to more definitively identify when the inhibited processes, be they singular or multiple, have returned to a state allowing for fertility.

Existing insights into possible biomarkers of RTF come from new evidence about molecular mechanisms involved in fertility-related processes and pathophysiology (110, 111) in addition to experimental measures of sub-fertility or infertility, and markers used to predict successful conception in *in vitro* fertilization (IVF) patients. FSH, AMH, and Inhibin B are examples of biomarkers falling in this latter category, and have been discussed above.

Depending on factors that qualify these molecular biomarkers as clinically applicable and directly or indirectly linked to successful conception, they may also be explored as a predictive marker for RTF following contraceptive method discontinuation. Potential biomarkers have been selected and scored (Table 1) on predefined criteria based on availability of a quantifiable human assay, the number and nature of potential sample sources, and whether their inhibition blocks associated fertility processes or there are any reports of marker alteration with contraceptive use that have been confirmed in other literature (Supplementary Table S1). The criteria we have selected for scoring attempts to determine analytically which candidates are most worth investigating from such a diverse and variable cohort. We have relied on factors we have defined as key to clinical viability, evidence that supports these prospective biomarkers’ intrinsic relationship with a specific fertility process which a contraceptive may act on, and if such action has already been demonstrated. This combination of benchmarks is well-rounded, is based on existing evidence, and could help guide future research more productively.

**Table 1:** Exploratory biomarkers of fertility associated processes with scored potential for prediction of return to fertility.

CT Method of Action	Marker	Original Source	Possible Detection Sources	Score*
Folliculogenesis	VEGF	Follicular Fluid	CVF (112), Serum (112)/Plasma (113), Saliva (114), Urine (115)	+ + + + +
	INSL3		Serum (116)/Plasma (117), Granulosa Cells	+ + +
	H2-relaxin		Serum (118)/Plasma (119), Granulosa Cells	+ + +
	Cx43	Granulosa Cells	Exosome (120)	+ + +
	KISS1	Cumulus Cells	Serum (121)/Plasma (122)	+ + +
	GDF-9	Follicular Fluid	Serum (123), Cumulus Cells	+ + +
	BMP-15		Serum/Plasma (123)	+ + +
	Endocannabinoids		Serum/Plasma (124), Fallopian Tubes (125)	+ +
Ovulation	PGE2	Cumulus Cells	Serum (126)/Plasma (127), Urine (128), Saliva (129)	+ + + + +
	MMPs	Granulosa Cells	Serum/Plasma (130), CVF (131), Urine (132), Saliva (133)	+ + + + +
	ADAMTS-1		Serum (134), Saliva (135), Urine (136)	+ + + + +
	LHR		–	+ + +
	PAPPA		Serum (137)/Plasma (138)	+ + +
	SERPINE2		Serum (139)/Plasma (140)	+ +
	Cystatin-S	Saliva	–	+ +
	VEGF	Follicular Fluid	CVF (112), Serum (112)/Plasma (113), Saliva (114), Urine (115)	+ + + + +
Endometrial transport and receptivity	Activin A	Uterine Fluid	Serum (141)/Plasma (142), Urine (143)	+ + + + +
	LIF	Endometrial Tissue	CVF (144), Serum (145)/Plasma (146)	+ + + + +
	*α*-inhibin		Serum (147)/Plasma (148)	
	Glycodelin-A		CVF (149), Serum (150)/Plasma (151)	+ + + +
	Mucins		CVF (152), CVM (153), Serum (154)/Plasma (155)	+ + + +
	Interleukin-18	Uterine Fluid	CVF (156), Serum (157)/Plasma (158), Saliva (159), Urine (160)	+ + +
	hDP		Serum (161), Menstrual Fluid (162)	+ + +
	Integrins	Endometrial Tissue	Serum (163), Urine (164)	+ + +
	BCL6		Serum/Plasma, Urine, Cervical Mucus (165)	+ + +
	Aromatase P450		–	+ +
	L-selectin ligand		Serum (166)/Plasma (167)	+ +
	Urocortin	Uterine Fluid	Serum (168)/Plasma (169)	+ +

VEGF, vascular endothelial growth factor; INSL3, insulin-like 3; GDF-9, growth differentiation factor 9; BMP-15, bone morphogenetic protein 15; Cx43, connexin 43; PGE2, prostaglandin E2; MMPs, matrix metalloproteinases; ADAMTS-1, a disintegrin and metalloproteinase with thrombospondin motif 1; PAPPA, pregnancy-associated plasma protein A; LHR, luteinizing hormone receptor; SERPINE2, serpin peptidase inhibitor, clade E, member 2; LIF, leukemia inhibitory factor; hDP, human decidua-associated protein; BCL6, B-cell lymphoma 6; CT, contraceptive method; CVF, cervicovaginal fluid.

* Score details are shown in the Supplementary Table.

Below we describe selected molecules, especially those that received higher relative scores within their category of contraceptive action, which may be further explored as biomarkers of RTF according to biological processes whose inhibition underpins mechanisms of contraceptive effect. The list of selected potential biomarkers is not comprehensive and all-inclusive; it merely brings attention to candidate markers that can be explored in relation to resumption of physiological reproductive processes associated with normal fertility. Many molecules intervene in more than one process and their classification under one category does not exclude them from others. Furthermore, a low score under our criteria results from a lack of current evidence for those factors which have been outlined above that we considered most relevant and promising for the development of biomarkers of RTF. It does not preclude the discovery of such evidence in the future that would lead to a more favorable re-evaluation of their promise.

### 5.1. Biomarker candidates associated with folliculogenesis

Contraceptive methods affecting the process of folliculogenesis, and RTF after discontinuation of methods thereof, may potentially be evaluated by markers associated with the development of the preovulatory follicle. There are a number of molecules involved in the maturation of the ovarian follicle that are readily obtainable and quantifiable, and thus may serve as biomarkers to be analyzed in contraceptive studies. Prime amongst them are relaxins and, in particular, insulin-like peptide 3 (INSL3). Relaxins are a family of peptide hormones known as neo-hormones, which are a class of paracrine and endocrine adaptations that relate to specific mammalian physiological functions, and are intimately involved with viviparity, placental development, and implantation, with their secretion typically occurring via the corpus luteum, ovarian follicles, and decidua (170). INSL3 has been shown to vary considerably across the menstrual cycle with episodic peaks in follicular and sometimes luteal phases, and is known to drop to undetectable levels in postmenopausal women (171). Relaxin-3 expression has been reported within oocytes in mid to late follicular stages of development, whereas receptor staining was localized to follicular cells (172). Further, it has been suggested that relaxin peptides may be involved in estradiol-dependent events in follicular development (172). Relaxin expression is also modified by medroxyprogesterone, RU-486, and glucocorticoids (173). H2-relaxin is another peptide hormone known specifically as a paracrine factor. Although H-2 relaxin has been identified primarily in the endometrium and corpus luteum, its gene is also constitutively expressed when granulosa cells have attained luteinized status under the effect of LH or hCG (174), making H2-relaxin a potential biomarker for the differentiation status of granulosa cells and thus follicular development. Additionally, oral contraceptives have been found to reduce H2-relaxin serum levels (175).

INSL3 specifically is an insulin-like peptide that is also part of the relaxin-like peptide family, and while it has been primarily considered a male hormone, it is also synthesized within the ovary. It is in part responsible for meiotic progression of oocytes in preovulatory follicles by increasing cAMP concentration through G protein-coupled receptor 8 (176). As a reproductive biomarker, INSL3 is relatively stable across the cycle of premenopausal women and very low or undetectable in postmenopausal women. Inverse correlation between INSL3 expression in theca cells of the antral follicles and atresia of granulosa cells has been observed (174). Of note sub-fertility and reduction in follicle numbers have been demonstrated in INSL3 knockout mice (177). INSL3 is quantifiable in serum and its levels have been associated with ovulatory and anovulatory cycles in adolescents (178).

Another potential biomarker of folliculogenesis is vascular endothelial growth factor (VEGF). Secretion of VEGF from granulosa and theca cells is correlated with increased angiogenesis of the ovarian follicle, increased vascularization coinciding with increased oocyte viability, and favorable pregnancy outcomes (179). Knockout of VEGF in female mice displayed not only decreased fertility and ovary size but a reduction in follicular development (180).

Another family of potential molecules of interest is the gap junction proteins called connexins. Cx43, for example, is associated with folliculogenesis and demonstrated to be elevated in growing granulosa cells but mostly absent in the corpus luteum and follicles undergoing atresia. A marked decrease in expression takes place following the LH surge in murine proestrus only in preovulatory follicles and associated cumulus cells, but not in more immature preantral follicles, establishing expression of Cx43 as a regulator and possible biomarker for follicular growth and development (181, 182).

Present in the hypothalamus and acting upon the hypothalamic-pituitary-ovarian axis, Kisspeptin (KISS1) is a neuropeptide lying upstream of gonadotropin-releasing hormone as an ovulatory trigger and has been experimentally identified to be associated with follicular development and oocyte maturation (183). Expression of KISS1 and its receptor have also been identified in human ovaries, including in theca cells, corpus luteum, cumulus cells, mural granulosa cells, and interstitial tissues and ovarian surface epithelium (184, 185). Conditional knockout of KISS1 and its receptor, GPR54, in mice leads to infertility and hypogonadotropic hypogonadism in addition to other maturation and reproductive system dysfunctions (186, 187).

Two members of the transforming growth factor-beta (TGFβ) superfamily highly expressed in oocytes and thought to affect oocyte function and development, GDF-9 and BMP-15, are promising candidates as well. Determination of GDF-9 levels in follicular fluid by Western blot analysis correlated higher levels with the nuclear maturation of oocytes via identification of the first polar body (188). GDF-9 mRNA expression has also been positively associated with oocyte maturation as well as fertilization and cleavage rates (189). BMP-15 is known to induce mitosis and proliferation in granulosa cells (188). Evaluation of BMP-15 levels in follicular fluid correlated higher concentrations with higher fertilization rate, cleavage, and embryo quality as defined by favorable morphology (189, 190). In knockout mice of both of these TGFβ members, subfertility and decreased ovulation have been demonstrated, but in GDF-9 knockouts specifically, cessation of folliculogenesis was also observed (191).

Endocannabinoids are lipid-signalling neurotransmitters with a broad dispersion and activity throughout the body. One specific endocannabinoid, anandamide, has been quantified in follicular fluid following oocyte aspiration during IVF treatment and was found to both be correlated to ovarian follicle size and maturity and to be synthesized by granulosa cells in developing follicles but not by oocytes, suggesting involvement in the antral phase of folliculogenesis (192, 193).

### 5.2. Biomarker candidates associated with cumulus expansion, follicular rupture, and ovulation

The mid-cycle LH surge in mammals sets in motion interconnected networks of signaling cascades to bring about rupture of the follicle and release of the oocyte during ovulation (194). Many mediators of these LH-induced signaling cascades are associated with inflammation, angiogenesis, steroidogenesis, and proteolysis, which act synergistically leading to a well-orchestrated process culminating in final oocyte maturation, follicle rupture and oocyte release. Novel contraceptive methods may alter molecular mechanisms mediating these processes and key molecules involved in them may be evaluated for prediction of RTF. A number of strong candidates for potential biomarkers can be found in this category.

Amongst the types of prostaglandins, PGE2 has been shown to be the most commonly involved in fertility and ovulation, with conditional knockouts suggesting its importance in the female reproductive system by demonstration of abnormal cumulus expansion, unruptured follicles, and subfertility when PGE2 or its receptor were targeted (195–199). Within the LH-induced signaling cascade, PGE2 has been shown to possess a specific role in cumulus expansion, inducing the upregulation of additional ovulatory genes mediated by cAMP and PKB-MAPK3/1 pathways (200). Additionally, antiestrogens and mifepristone have been shown to decrease uterine concentrations of PGE2 (201), while levonorgestrel decreased multiple prostaglandins, including PGE2 in a dose dependent manner (202).

Matrix metalloproteinases (MMPs) are a family of proteases primarily capable of degrading extracellular matrix (ECM) proteins. Members of this family are posited to regulate the complex system of endometrial connective tissue turnover and remodeling throughout the menstrual cycle, exhibiting a large degree of control over ovarian physiology (203). Expression of MMP has been shown to be induced by an LH or hCG surge, implicating it in the process of ovulation and follicular rupture (204). More evidence pointing to the role of MMPs in this process is the inhibition of ovulation by the metalloproteinase inhibitor GM6001 of rhesus macaque follicles inducing abnormality or absence of the normal stigmata indicative of follicle rupture (205). MMP10 specifically is a stromelysin theorized to potentially be involved in ECM modulation during follicular remodeling and regulating vascularity in the theca and granulosa cells. The regulation of MMP-10 has been studied in humans and rats and determined to be mediated by progesterone and EGF receptor signaling pathways, playing an important role in ovulation and luteinisation (206).

ADAMTS-1 is a disintegrin and metalloproteinase (ADAM) with thrombospondin type 1 motifs expressed in a wide variety of accessible bodily fluid sources. Examination of cumulus granulosa cells in women undergoing IVF revealed correlation between ADAMTS-1 expression and successful fertilization of denuded oocytes, with a three-fold higher expression rate of ADAMTS-1 in successfully vs. unsuccessfully fertilized oocytes (207). ADAMTS-1 expression has been shown to peak at a period 12 h after a simulated LH surge, when follicular rupture begins, and to be linked to progesterone receptor, the deficiency of which causes both under expression of ADAMTS and failure of follicle wall rupture (208). It has been demonstrated to be related to histological regulation of the uterus and ovaries and fertilization, with ADAMTS-1 null female mice ovulating only one-ninth of the total number of oocytes (209), as well as displaying impaired follicular development, implantation, and intrauterine development more broadly (210).

Luteinizing hormone receptor (LHR) mediates the binding action of LH, a key regulator of oocyte maturation and ovulation. LHR mRNA expression has been investigated in luteinized granulosa cells obtained from aspirated preovulatory follicles in women undergoing IVF, correlating a higher expression rate in cumulus cells with decreased fertilization rate (211). LHR gene expression was also found, however, to be higher in larger and more mature oocytes, to rise during antral follicular growth, and to be high in mural granulosa cells, preceding ovulation (211). Levels of LHR could be a marker of oocyte maturation and readiness for cumulus expansion.

Pregnancy-associated plasma protein-A (PAPPA) is a protease with an IGFBP substrate associated with hormone modulation found in cumulus granulosa cells. mRNA levels of PAPPA have been shown to be elevated both in more mature oocytes and those that resulted in live birth vs. those that did not (212). Additionally, PAPPA knockout mice were shown to have reduced ovulated oocytes, likely related to associated alteration of IGFBP4 proteolytic activity (213).

Some weaker but still promising candidates include serine protease inhibitor E2 (SERPINE2), which has been correlated with oocyte maturity and successful fertilization (214), and Cystatin S, isoforms of which were found to be heavily isolated to ovulatory vs. pre- or post-ovulatory phases (215, 216).

### 5.3. Biomarker candidates associated with endometrial transport and receptivity

Endometrium plays a dynamic role throughout the menstrual cycle to allow for adequate gamete transport, fertilization and successful embryo implantation. These changes are governed by progesterone and estrogen responsive molecular pathways and involve several processes leading to cell differentiation and tissue remodeling. Impairment or dysregulation of the processes and mechanisms leads to pathology and infertility (217). In healthy fertile women, an array of cytokines, growth factors, transcription factors, cell adhesion molecules, and prostaglandins have been described during the receptive phase (218). The molecular events associated with the window of embryo receptivity include the expression of decidualization markers like prolactin and Insulin-like growth factor binding proteins (IGFBPs) (219). Additionally, the decidual stromal cells exhibit upregulation of homeobox proteins (HOX)A10, HOXA11, and leukemia inhibitory factor (LIF), which are signature biomarkers for endometrial receptivity.

Showing promise in tracking endometrial transport and receptivity, VEGF has also been identified as a variably expressed factor in the endometrium across the menstrual cycle. A peak in the mid-luteal phase of endometrial VEGF expression, as well as impaired levels in infertile patients during the same phase, signify a role in embryo implantation (220, 221). A decrease in VEGF expression in endometrial stromal cells has been demonstrated after administration of mifepristone (222) as well as oral desogestrel/ethinyl estradiol and etonogestrel implant (223). However, a levonorgestrel-releasing intrauterine system has been implicated in elevated endometrial tissue levels of VEGF mRNA (224) and certain isoforms were also elevated by medroxyprogesterone acetate, norethindrone, and levonorgestrel in Ishikawa cell lines (225).

There are two additional strong candidates for endometrial transport and receptivity biomarkers. Activin A is a dimeric multifunctional growth factor of the transforming growth factor beta (TGF-ß) family that plays a key role in endometrial receptivity, trophoblast invasion, and embryo implantation (226). Additionally, Activin A has been linked to the production of ovarian steroid hormones, oocyte maturation, and follicular development (227) and mice deficient in activin receptors showcase reproductive deficiency and suppression of follicle-stimulating hormone (228). The role of Activin A as a possible biomarker of fertility and implantation success has been supported by elevated levels found in endometrial washing fluid of women who subsequently became pregnant after intrauterine insemination vs. those who did not, as measured by ELISA (229). A counterpart to activin, *α*-inhibin is also a member of the TGF-ß and has a negative regulatory effect upon actin. An increase of *α*-inhibin gene expression in the endometrium during the mid-secretory phase of the menstrual cycle was correlated with higher rates of implantation failure vs. confirmed clinical pregnancies in women undergoing IVF and embryo transfer (230). Alteration of expression by progestin-only contraceptives has been demonstrated for both Activin A and related *α*-inhibin subunits at both the protein and mRNA level, as well as during the menstrual cycle and early pregnancy (231).

Endometrial LIF, a cytokine of the interleukin 6 family, is another such promising marker. Differences in expression in the secretory phase between fertile and infertile women are demonstrably striking (232) with immunostaining of endometrial biopsy samples finding strong LIF expression to yield a 6.4-fold higher pregnancy rate than weaker intensities in women undergoing IVF (233). Embryo attachment failure in mice following treatment with levonorgestrel, at a dose established to be equivalent to use as emergency contraceptives in humans, has been observed alongside significantly reduced uterine LIF expression (234). However, it should be noted that evaluations of the effect of emergency contraceptive levonorgestrel in humans generally does not support a demonstrable effect on endometrial receptivity and some other associated markers (235).

Glycodelin is a glycoprotein of the lipocalin superfamily present in the uterus as a secretion of decidualized endometrial glands and stromal cells and is a proposed biomarker for endometrial function (236). Glycosylated isoforms have been shown to be differentially expressed across phases of the menstrual cycle, with an altered profile of expressed glycoforms in endometriosis during the use of intrauterine devices, an evaluation which supports the potential of GDA as a biomarker of endometrial receptivity (237). Other potential markers whose expression is altered during use and after removal of IUDs include LIF, IGFBP family of proteins, keratin 8, folate receptor, and MMP10 (238).

The mucus in the cervical canal controls the ascent of spermatozoa and is a target of contraceptive action by progestin-only pills and levonorgestrel (89–91, 239, 240). A variety of mucins are expressed on the epithelia of the upper genital tract and have been posited to act as inhibitors of implantation, although examinations of the exact role of each, and of the more frequently studied MUC1 specifically, show some contrasting results (241–243). The membrane-bound MUC16 measured in endometrial samples correlated lower expression with recurrent pregnancy loss and conception after IVF, and higher expression with elevated progesterone during ovarian stimulation (244). MUC16 has been proposed as a potential marker for fertility during normal menstrual cycling, acting as a regulator of the local osmotic modulus and mucus layer integrity in the reproductive tract (152). Amongst the gel-forming mucins identified in humans, MUC2, MUC5AC, MUC5B and MUC6, MUC2 related polymorphisms have been related to infertility and endometriosis (245). MUC2 has also shown significant differences in expression under the effects of oral contraceptives (246). Some additional candidates identified that proved weaker under our specific scoring criteria but remain somewhat promising for predicting endometrial receptivity include Interleukin-18, hDP, Integrins, BCL6, Aromatase P450, L-selectin ligand, and urocortin.

There exist several limitations constraining effective clinical implementation of prospective biomarkers in tandem with experimental validation. Regulatory requirements that seek to verify certain standards of clinical utility can require an extensive process of testing and justification that contain any number of stumbling blocks for potential marker candidates. Specificity, sensitivity, and accuracy at predicting interruption and RTF need to be established before considering candidate molecules as true biomarkers. Providing or developing an assay method which meets standard for clinical utilization with regards to quality control and assurance, speed, and sample storage or transportation from readily accessible sources is a challenge in and of itself beyond the hurdles for proving analytical performance and clinical relevance in decision making. The FDA requires a multi-step process by which these factors are analyzed through their Biomarker Qualification Program (247).

## 6. Discussion

Further exploration of potential new, reproducible biomarkers for effective evaluation of RTF is necessary to complement and bring full circle the promise of new contraceptive products and methods. Predictive biomarkers that also pass the hurdles of easy, viable clinical utility and low cost could potentially supply users of not only existing contraceptive products but also those looking to incorporate new methods into their family planning choices with the information necessary to confidently make those selections, increasing uptake and method continuation.

Historically, the main indicator for evaluating RTF has been fecundity as defined by achievement of pregnancy or birth. For contraceptives based on various types and combinations of steroid hormones with the primary mechanisms of action being suppression of ovulation, evaluating RTF following discontinuation of use has generally combined data on contraceptive steroid levels declining to undetectable and data on ovulation based on measuring progesterone levels as a surrogate. Some studies have more rigorously and accurately assessed ovulation using Hoogland scores which add vaginal ultrasound of follicle development/rupture along with progesterone. Since the latter studies are more challenging to conduct, characterizing progesterone alone using several different protocols has been most commonly used. Additionally, there are diverse interindividual and variable factors like pharmacogenomic, BMI, ethnicity, and steroid response differences to consider when attempting to derive a meaningful RTF evaluation from resumption of ovulation, even when it is the main mechanism in question. Secondary mechanisms beyond the direct suppression of ovulation involving the regulation of folliculogenesis, cumulus expansion, and oocyte maturation for example, and any associated inadequacy of the luteal phase or luteinized unruptured follicle, as well as alterations to cervical mucus and endometrium, are implicated mechanisms in progestin dominated methods such as POPs and LNG implants, which may confound the interpretation of ovulation related markers of RTF.

Based on the overview of potential biomarkers of RTF summarized above, it is clear that opportunities exist to add alternative markers to the current surrogate endpoints of return to ovulation. Furthermore, combining exploratory and existing biomarkers would also assist in evaluating RTF for methods targeting more than the direct suppression of ovulation. Additional research determining the association between, and predictive value of, these biomarker candidates and RTF after contraceptive discontinuation is needed. Clinical validation studies should include various existing contraceptives and known endpoints of RTF after contraceptive discontinuation such as follicle growth, ovulation, and cyclical endogenous hormones and endometrial changes, or pregnancy. As a starting point, one adequately designed study with different types of contraceptives may include these “gold standards” and the assessment of multiple potential biomarkers reflecting the above categories. Based upon association with potential mechanisms of contraceptive action, demonstrable detection in plasma or CVF, and *in vivo* evidence of alteration by existing contraceptives that contribute to higher scores in Table 1, we propose the following candidate molecules as potential markers of fertility associated processes to be measured at multiple timepoints during and after contraceptive use and discontinuation: VEGF, H2-relaxin, PGE2, MMP10, Activin A, LIF, MUC2, and MUC16. Representing known and potential targets for contraception, these molecules may provide new tools to assess interruption and resumption of physiological processes involved in fertility and could be evaluated in a clinical trial that assesses multiple timepoints during use and following discontinuation of various contraceptives.

Considerable testing and validation need to be performed in order to move any of the biomarkers proposed from the bench to use in a clinical setting. Key considerations include accuracy and validation of assays for the factors of interest (248). Additionally duration of follow-up, the invasiveness of procedures for sampling, and reproducibility, are factors of exceptional importance when moving from the bench to clinical validation (248) in order to demonstrate whether the biomarker would meet regulatory requirements and have a potential clinical benefit. Ideally, approaches to assess RTF following discontinuation of a contraceptive would be simple, easily-implementable, non-invasive, sensitive, specific, and present a low burden for users and for health care providers.

We recognize that many of the proposed molecules individually are not specific to reproductive processes, and therefore determination of associated and comparative levels and thresholds will be necessary to provide more precise information about sensitivity and specificity as they relate to fertility, particularly the resumption of fertility following contraception. A flexible combination of molecules may also provide a more informative assessment mitigating potential concerns about non-specificity, differing subgroups, or new contraceptives utilizing multiple and varied mechanisms of action in the context of analyzing RTF. More studies are clearly warranted before these candidate molecules can become biomarkers.

In this review, we have not considered the influence endocrine-disrupting chemicals (EDCs), which are ubiquitous and have been associated with disruption of ovarian cyclicity, reduced fertility, infertility, and other reproductive disruptions (249–251). It is possible that unmeasured exposure to prevalent EDCs could have influenced differences seen in RTF across many studies summarized. In addition, widely consumed phytoestrogens (natural plant EDCs including soy) may have effects on fertility/fecundity (252). Given mounting evidence of the influence of multiple different EDCs on the reproductive system, consideration should be given to measuring select EDCs known to influence reproductive function in future RTF research.

This review intends to bring attention to a gap in the knowledge related to prediction of RTF after new and existing contraceptive use and discontinuation. Current biomarkers rely on return to ovulation or its predictive hormonal changes. However multiple or new mechanisms of contraceptive action are not adequately evaluated by these markers. A series of molecules that might be explored and further considered as potential biomarkers of RTF following use of existing and new contraceptives are proposed. After validation, these biomarkers may be able to further assist in predicting the ability of women to conceive after contraceptive discontinuation, especially following use of new contraceptives that do not rely on suppression of ovulation as a main mechanism of action. Further research is warranted to identify the most promising of the list of candidates and subsequently validate them clinically.
