# Supplementary material for: Potential biomarkers to predict return to fertility after discontinuation of female contraceptives—looking to the future
Source: Front Reprod Health. 2023 Aug 22;5:1210083. doi: 10.3389/frph.2023.1210083 (PMC10477712; doi:10.3389/frph.2023.1210083)
Supplement: Supplementary file 1 [file Table1.docx]

Supplemental Table 1. Criteria and scoring of potential biomarkers of return to fertility

| **Marker** | Quantifiable in human samples by known assay | Type of assay | Bodily Fluid source (plasma/serum) | Additional Bodily Fluid Source (CVF, Urine, Saliva) | Inhibition blocks fertility process | Expression altered by contraceptives | Total |
| --- | --- | --- | --- | --- | --- | --- | --- |
| VEGF | + | ELISA, Immunohistochemistry | + | + | +^(^[^121^](#_ENREF_121)^)^ | +_A,B_^(^[^163^](#_ENREF_163)^,^ [^164^](#_ENREF_164)^,^ [^166^](#_ENREF_166)^)^ | +++++ |
| INSL3 | + | ELISA, Radioimmunoassay, qPCR | + |  | +^(^[^118^](#_ENREF_118)^)^ |  | +++ |
| H2-relaxin | + | ELISA | + |  |  | +_B_^(^[^114^](#_ENREF_114)^)^ | +++ |
| Cx43 | + | qPCR |  |  | +^(^[^256^](#_ENREF_256)^)^ | +_B_^(^[^257^](#_ENREF_257)^)^ | +++ |
| KISS1 | + | ELISA, qPCR, Immunohistochemistry | + |  | +^(^[^127^](#_ENREF_127)^)^ |  | +++ |
| GDF-9 | + | ELISA, Western Blot, qPCR | + |  | +^(^[^132^](#_ENREF_132)^)^ |  | +++ |
| BMP-15 | + | ELISA, Western Blot, qPCR | + |  | +^(^[^132^](#_ENREF_132)^)^ |  | +++ |
| Endocannabinoids | + | Isotope-dilution LC-MS, UHPLC-MS/MS | + |  |  |  | ++ |
| PGE2 | + | ELISA | + | + | +^(^[^136-138^](#_ENREF_136)^)^ | +_B_^(^[^143^](#_ENREF_143)^)^ | +++++ |
| MMPs | + | ELISA | + | + | +^(^[^146^](#_ENREF_146)^)^ | +_A_^(^[^258^](#_ENREF_258)^)^ | +++++ |
| ADAMTS-1 | + | ELISA, qPCR | + | + | +^(^[^149^](#_ENREF_149)^)^ | +_A_^(^[^259^](#_ENREF_259)^)^ | +++++ |
| LHR | + | ELISA, qPCR |  |  | +^(^[^261^](#_ENREF_261)^)^ | +_A_^(^[^262^](#_ENREF_262)^,^ [^263^](#_ENREF_263)^)^ | +++ |
| PAPPA | + | ELISA, qPCR | + |  | +^(^[^260^](#_ENREF_260)^)^ |  | +++ |
| SERPINE2 | + | ELISA, qPCR | + |  |  |  | ++ |
| Cystatin-S | + | ELISA, qPCR |  | + |  |  | ++ |
| Activin A | + | ELISA | + | + | +^(^[^169^](#_ENREF_169)^)^ | +_A,B_^(^[^163^](#_ENREF_163)^,^ [^172^](#_ENREF_172)^)^ | +++++ |
| LIF | + | ELISA, IHC Staining | + | + | +^(^[^264^](#_ENREF_264)^)^ | +_A_^(^[^175^](#_ENREF_175)^)^ | +++++ |
| α-inhibin | + | ELISA, qPCR | + |  | +^(^[^266^](#_ENREF_266)^)^ | +_A_^(^[^172^](#_ENREF_172)^,^ [^267^](#_ENREF_267)^)^ | ++++ |
| Glycodelin-A | + | ELISA | + | + |  | +_A_^(^[^265^](#_ENREF_265)^)^ | ++++ |
| Mucins | + | ELISA | + | + |  | +_B_^(^[^163^](#_ENREF_163)^)^ | ++++ |
| Interleukin-18 | + | ELISA | + | + |  |  | +++ |
| hDP | + | ELISA, IF | + | + |  |  | +++ |
| Integrins | + | ELISA, IHC Staining | + | + |  |  | +++ |
| BCL6 | + | ELISA, IHC Staining | + | + |  |  | +++ |
| Aromatase P450 | + | ELISA |  |  | +^(^[^268^](#_ENREF_268)^)^ |  | ++ |
| L-selectin ligand | + | ELISA, Western Blot | + |  |  |  | ++ |
| Urocortin | + | ELISA | + |  |  |  | ++ |

A—Demonstrated in vivo

B—Demonstrated in vitro

VEGF: vascular endothelial growth factor; INSL3: insulin-like 3; GDF-9: growth differentiation factor 9; BMP-15: bone morphogenetic protein 15; Cx43: connexin 43; KISS1: Kisspeptin; PGE2: prostaglandin E2; MMPs: matrix metalloproteinases; ADAMTS-1: a disintegrin and metalloproteinase with thrombospondin motif 1; PAPPA: pregnancy-associated plasma protein A; LHR: luteinizing hormone receptor; SERPINE2: serpin peptidase inhibitor, clade E, member 2; LIF: leukemia inhibitory factor; hDP: human decidua-associated protein; BCL6: B-cell lymphoma 6; ELISA: enzyme-linked immunosorbent assay; qPCR: quantitative polymerase chain reaction; LC-MS: liquid chromatography-mass spectrometry; IHC: immunohistochemistry
